# Relationship between cognitive function and weight-adjusted waist index in people ≥ 60 years old in NHANES 2011–2014

**DOI:** 10.1007/s40520-023-02649-8

**Published:** 2024-02-09

**Authors:** Xue-li Wang, Hong-lin Feng, Xiao-zhuo Xu, Jing Liu, Xu Han

**Affiliations:** 1 First Clinic Medical School, Nanjing University of Chinese Medicine, Nanjing, China; 2 Geriatric Department, Affiliated Hospital of Nanjing University of Chinese Medicine, 155 Hanzhong Road, Nanjing, China

**Keywords:** Cross-sectional study, WWI, Cognitive function, NHANES, Older adult

## Abstract

**Background:**

Widespread attention has been given to the detrimental effects of obesity on cognitive function. However, there is no evidence on the connection between low cognitive performance and the WWI (weight-adjusted waist index). This study looked into the connection between poor cognitive performance and the WWI in senior Americans.

**Methods:**

A cross-sectional research study was carried out with information from the NHANES 2011–2014. With multivariate linear regression models, the pertinence between the WWI and low cognitive function in persons older than 60 years was examined. The nonlinear link was described using threshold effect analyses and fitted smoothed curves. Interaction tests and subgroup analysis were also conducted.

**Results:**

The study had 2762 individuals in all, and subjects with higher WWI values were at greater risk for low cognitive function. In the completely adjusted model, the WWI was positively connected with low cognitive performance assessed by CERAD W-L (OR = 1.22, 95% CI 1.03–1.45, *p* = 0.0239), AFT (OR = 1.30, 95% CI 1.09–1.54, *p* = 0.0029), and DSST (OR = 1.59, 95% CI 1.30–1.94, *p* < 0.0001). The effect of each subgroup on the positive correlation between the WWI and low cognitive performance was not significant. The WWI and low cognitive performance as determined by CERAD W-L and AFT had a nonlinear connection (log-likelihood ratio < 0.05).

**Conclusion:**

Among older adults in the United States, the risk of low cognitive performance may be positively related to the WWI.

## Introduction

Population aging is a global phenomenon, with 2.1 billion people worldwide expected to be over 60 years old by 2050 [1]. At the same time, the number of older people with dementia is increasing rapidly. Alzheimer’s dementia currently affects an estimated 6.7 million older persons in the US, and as of 2019, it has become the sixth most common cause of mortality in the US [2, 3]. As an important risk factor for various neurodegenerative diseases including dementia [4–6], cognitive decline is gradually gaining attention and is increasingly becoming a necessary topic in medical consultations.

Obesity is a new but complicated risk factor for dementia and poor cognitive function, especially in older persons [7, 8]. Some studies suggest that excessive obesity can lead to cognitive decline and dementia [9–12], while others suggest that body fat is protective against poor cognitive function and dementia in elderly persons [13–16]. This may be due to the effects of age on the distribution regions of adiposity. In the relation between obesity and low cognitive performance, the area of distribution of obesity(e.g., central or overall) may be important [17]. However, some common obesity measurement indices, like body mass index (BMI), lack sensitivity in identifying body fat distribution [18, 19]. Based on this, a new index for assessing obesity called the WWI (weight-adjusted waist index), has been proposed to evaluate obesity by weight-standardized waist circumference. The WWI can reflect weight-independent central obesity and has better accuracy than BMI. Related studies suggest that the WWI may indicate changes in fat and muscle components associated with aging-related changes and is universally applicable to all races [20–22]. This study showed that an increase in WC (waist circumference) was positively connected to a higher likelihood of poor cognitive function after adjustment for BMI [23, 24].

Currently, most studies linking obesity to cognitive decline fail to consider how fat distribution relates to cognitive decline. Therefore, the researchers aimed to assess any potential links between the WWI and poor cognition using information from the National Health and Nutrition Examination Survey (NHANES). The authors speculate that a higher WWI is possibly connected to a higher risk of poor cognition.

## Methods

### Survey description

The NHANES survey provided the data, which is a national population-based cross-sectional survey conducted by the National Center for Health Statistics (NCHS) to examine the nutrition and health status of Americans [25]. NHANES is a cross-sectional survey with a two-year cycle that uses typical samples and complicated multistage stratified random sampling. Participants underwent a home interview initially, followed by a mobile examination center (MEC)-based health examination [26]. The study protocol was evaluated and approved by the NCHS Research Ethics Review Board, and written informed permission was acquired from each participant.

### Study population

**Fig. 1:**
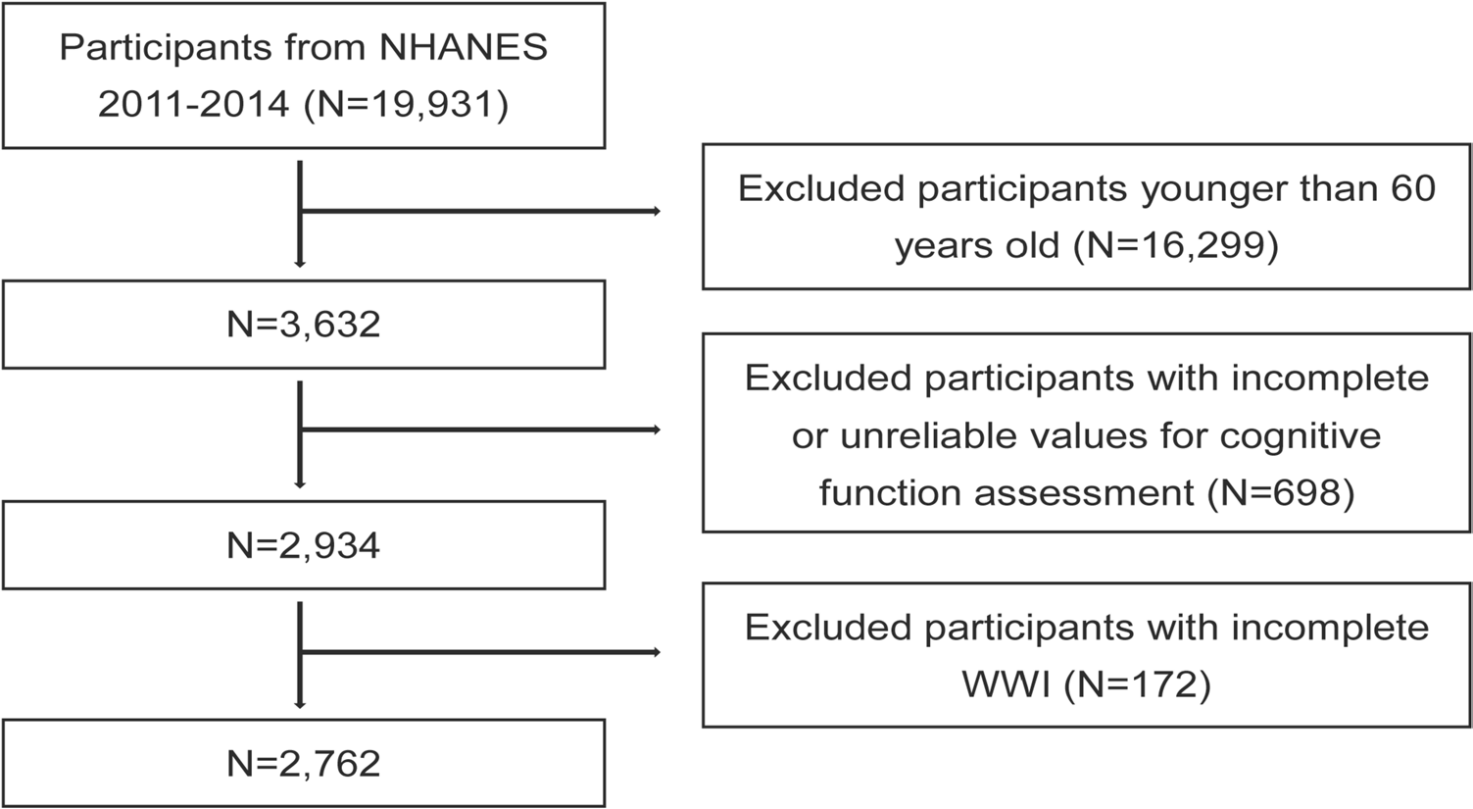
The participant selection flowchart

This study included data from two NHANES cycles, 2011–2012 and 2013–2014, with information on cognitive function measures and complete variables (weight, WC) to calculate WWI. A total of 19,931 individuals were enrolled in NHANES from 2011 to 2014, and our research was restricted to adults aged 60 and over who completed the MEC Cognitive Functioning Survey (*n* = 3632). Then, those with incomplete WWI (*n* = 172) and people with incomplete cognitive tests or inaccurate scores on the three cognitive function measures (*n* = 698) were excluded. After the exclusions, 2762 individuals were included in this research (Fig. 1).

### WWI assessment

The WWI (weight-adjusted waist index) is a new index for the evaluation of human obesity that is proportional to age and reflects physical alterations associated with age. The formula for WWI (cm/√kg) was calculated as WC (cm) divided by the square root of weight (kg), and was derived from WC and standardized based on body weight [21]. The body measurement data regarding weight and WC were obtained by certified health technicians associated with the NHANES. In the study, the WWI was selected as the exposure variable.

### Cognitive function assessment

This study used three tests to assess cognitive function: the Centre for the Establishment of a Registry for Alzheimer’s Disease (CERAD) Word Learning Score Test (CERAD W-L), the Animal Fluency Test (AFT) and the Digit Symbol Substitution Test (DSST) [27]. The CERAD W-L tests episodic long-term memory for verbal material and is composed of three sequential learning trials (Immediate Recall Test, IRT) and a delayed recall test (DRT), with the total mark for all four tests ranging from 0 to 40 points. The AFT primarily assesses the lexical-semantic component of the language domain and concurrently assesses executive functions [28], with scores ranging from 3 to 39. The Wechsler Adult Intelligence Scale-III (WAIS-III) performance module is called DSST, which gauges visuoperceptual functions (including scanning, writing and drawing), sustained attention, and processing speed [29], with scores in the range of 0 to 105. Lower test scores indicate poorer cognitive function.

The cognitive function testing outcomes including CERAD W-L, AFT, and DSST were treated as outcome variables. There is not yet a gold standard cutoff point for the results of the CERAD W-L, AFT, and DSST. However, earlier studies using the NHANES database used the lowest quartile of test scores as the cutoff point [30]. In this study, those who participated with test scores below the quartile were considered cognitively dysfunctional, with cutoff values of 21 for the CERAD W-L, 13 for the AFT, and 34 for the DSST.

### Selection of covariates

We incorporated the covariates connected to WWI or cognitive function from earlier research and excluded collinearity issues, including age (years), sex (male/female), race (Mexican American/other Hispanic/non-Hispanic White/non-Hispanic Black/Other race), education level (below high school/high school/above high school), marital status (cohabitation: married/living with someone/solitude: widowed/divorced/separated/unmarried), PIR (Poverty–income ratio; ≤ 1: below the poverty line, > 1: above the poverty line) [31], BMI (body mass index, kg/m2), smoking status (yes/no), alcohol consumption (yes/no), hypertension (yes/no) and diabetes (yes/no). All information was acquired by standardized questioning, physical examination and laboratory tests, and questionnaires were provided by qualified staff in the medical field.

### Statistical analysis

According to CDC guidelines, statistical analysis was carried out with proper NHANES sample weights while taking into consideration complicated multistage cluster surveys. When 2 2-year cycles of continuous data were combined, a fresh sample weight was calculated by dividing the previous 2-year sample weight by 2 [32]. In the baseline characteristics table, standard deviations (SDs) are used to describe continuous data, and proportions are used to depict categorical variables. To test for differences between the poor and normal cognitive function groups, the weighted Student’s t test was used for continuous variables and the weighted Chi-square test was used for categorical variables. We analyzed the correlation between the WWI and low cognitive performance using multivariate logistic regression methodology, and three models were used to evaluate the relationship. In Model 1, no covariates were adjusted. Age, sex and race were adjusted for in Model 2. and Model 3 was adjusted for sex, age, race, education level, marital status, PIR, BMI, smoking status, alcohol consumption, hypertension and diabetes. [33] Given that the percentage of missing data was less than 10% of the overall sample, no imputation was performed. We further analyzed the WWI after dividing it into quartiles to evaluate the robustness of the correlations. Subgroup analysis of the relevance between the WWI and low cognitive performance was conducted to assess the stability of the correlation. All findings from multiple logistic regression and subgroup analyses were analyzed using the results from Model 3. The impacts of WWI on low cognitive performance were examined using smooth curve fitting to determine if there were any nonlinear relationships and calculate the threshold effect. The statistical software packages R (The R Foundation; http://www.r-project.org; version 4.1.3) and EmpowerStats (www.empowerstats.com; X&Y Solutions Inc.) were used for all analyses. Statistical significance was set as α = 0.05.

## Results

### Baseline characteristics of participants

Our research included 1353 males and 1409 females from NHANES (2011–2014). The average age of all involved patients was 69.28 ± 6.74 years, and the average WWI was 11.49 ± 0.71. The CERAD W-L, AFT, and DSST showed that 23.97%, 22.85%, and 24.55% of the participants had low cognitive function, respectively. The sociodemographic information of the respondents was classified and described according to age, sex, race, educational level, marital status, PIR, BMI, smoking status, alcohol consumption, hypertension, and diabetes. Based on three tests related to cognitive performance (CERAD W-L, AFT, DDST), there were notable variations in the distributions of age, race, education level, PIR, hypertension and the WWI between those with low and normal cognition. Compared with those with normal cognition, those who had poor cognition were more likely to be older, less educated, and poorer in terms of income, and have a higher WWI, and prevalence of hypertension. On the DSST and AFT, the prevalence of diabetes was markedly higher in individuals with poor cognition than in individuals with normal cognition, and the rate of alcohol consumption was lower than in those with normal cognition. On the CERAD W-L and DSST, those who had poor cognitive function were more likely to be male, while those with normal cognition were more likely to be female. Detailed data are displayed in Table 1.

**Table 1:** NHANES 2011–2014 study population characteristics

Variables	CERAD W-L			AFT			DDST		
	Normal cognitive performance (*n* = 2100)	Low cognitive performance (*n* = 662)	*p* value	Normal cognitive performance (*n* = 2131)	Low cognitive performance (*n* = 631)	*p* value	Normal cognitive performance (*n* = 2084)	Low cognitive performance (*n* = 678)	*p* value
Age (years)*	68.48 ± 6.47	71.79 ± 6.95	< 0.001	68.79 ± 6.61	70.92 ± 6.91	< 0.001	68.65 ± 6.59	71.20 ± 6.83	< 0.001
BMI (kg/m^2^)*	29.16 ± 6.25	28.40 ± 6.05	0.006	29.05 ± 6.22	28.74 ± 6.19	0.278	29.06 ± 6.28	28.74 ± 6.01	0.253
*Sex*, *n* (%)			< 0.001			0.778			< 0.001
Male	942 (44.86)	411 (62.08)		1047 (49.13)	306 (48.49)		973 (46.69)	380 (56.05)	
Female	1158 (55.14)	251 (37.92)		1084 (50.87)	325 (51.51)		1111 (53.31)	298 (43.95)	
*Race*, *n* (%)			0.003			< 0.001			< 0.001
Mexican American	174 (8.29)	75 (11.33)		197 (9.24)	52 (8.24)		156 (7.49)	93 (13.72)	
Other Hispanic	201 (9.57)	87 (13.14)		208 (9.76)	80 (12.68)		158 (7.58)	130 (19.17)	
Non-Hispanic White	1011 (48.14)	293 (44.26)		1102 (51.71)	202 (32.01)		1121 (53.79)	183 (26.99)	
Non-Hispanic Black	496 (23.62)	155 (23.41)		429 (20.13)	222 (35.18)		421 (20.20)	230 (33.92)	
Other race	218 (10.38)	52 (7.85)		195 (9.15)	75 (11.89)		228 (10.94)	42 (6.19)	
*Education level*, *n* (%)			< 0.001			< 0.001			< 0.001
Below high school	145 (6.91)	158 (23.90)		176 (8.26)	127 (20.19)		73 (3.50)	230 (34.02)	
High school	268 (12.77)	123 (18.61)		254 (11.92)	137 (21.78)		228 (10.94)	163 (24.11)	
Above high school	1686 (80.32)	380 (57.49)		1701 (79.82)	365 (58.03)		1783 (85.56)	283 (41.86)	
*Marital Status*, *n* (%)			0.278			0.002			< 0.001
Cohabitation	1241 (59.18)	376 (56.80)		1282 (60.22)	335 (53.17)		1279 (61.46)	338 (49.85)	
Solitude	856 (40.82)	286 (43.20)		847 (39.78)	295 (46.83)		802 (38.54)	340 (50.15)	
*PIR*, *n* (%)			< 0.001			< 0.001			< 0.001
≤ 1	294 (15.26)	133 (22.06)		281 (14.33)	146 (25.70)		240 (12.51)	187 (30.61)	
> 1	1632 (84.74)	470 (77.94)		1680 (85.67)	422 (74.30)		1678 (87.49)	424 (69.39)	
*Smoking status*, *n* (%)			0.549			0.890			0.154
Yes	1059 (50.48)	343 (51.81)		1083 (50.87)	319 (50.55)		1042 (50.02)	360 (53.18)	
No	1039 (49.52)	319 (48.19)		1046 (49.13)	312 (49.45)		1041 (49.98)	317 (46.82)	
*Alcohol consumption, n* (%)			0.105			< 0.001			< 0.001
Yes	1773 (85.08)	535 (82.43)		1814 (85.93)	494 (79.42)		1782 (86.09)	526 (79.34)	
No	311 (14.92)	114 (17.57)		297 (14.07)	128 (20.58)		288 (13.91)	137 (20.66)	
*Hypertension*, *n* (%)			0.021			< 0.001			< 0.001
Yes	1406 (66.95)	475 (71.75)		1409 (66.12)	472 (74.80)		1360 (65.26)	521 (76.84)	
No	694 (33.05)	187 (28.25)		722 (33.88)	159 (25.20)		724 (34.74)	157 (23.16)	
*Diabetes, n* (%)			0.144			< 0.001			< 0.001
Yes	539 (25.68)	189 (28.55)		527 (24.73)	201 (31.90)		494 (23.70)	234 (34.56)	
No	1560 (74.32)	473 (71.45)		1604 (75.27)	429 (68.10)		1590 (76.30)	443 (65.44)	
WWI (cm/√kg)*	11.46 ± 0.71	11.57 ± 0.71	< 0.001	11.46 ± 0.70	11.58 ± 0.74	< 0.001	11.44 ± 0.70	11.63 ± 0.74	< 0.001

*Data are presented as the mean ± standard deviation

### Association between the WWI and cognitive function

Table 2 displays the correlation between the WWI and poor cognition. The current research indicated that an increased risk of poor cognitive function was linked to an increased WWI. In Model 1, a positive relationship was observed between the WWI and poor cognition related to CERAD W-L (OR = 1.22, 95% CI 1.03–1.45, *p* = 0.0239), AFT (OR = 1.30, 95% CI 1.09–1.54, *p* = 0.0029), and DSST (OR = 1.59, 95% CI 1.30–1.94, *p* < 0.0001), indicating that an elevated risk of poor cognition is associated with an increased WWI. The WWI was further converted into a categorical variable (quartiles), which was used to judge the robustness of the correlations. The findings showed that on the AFT and DSST tests, the risk of poor cognitive function becomes greater as WWI rises compared to the group with the lowest WWI (P for trend < 0.05).

**Table 2:** Link between the WWI and low cognitive performance

Model 1	CERAD W-L		AFT		DDST	
	OR (95% CI)	*p* value	OR (95% CI)	*p* value	OR (95% CI)	*p* value
WWI	1.22 (1.03, 1.45)	0.0239	1.30 (1.09, 1.54)	0.0029	1.59 (1.30, 1.94)	< 0.0001
*WWI (quartile)*						
Q1	Ref		Ref		Ref	
Q2	0.98 (0.73, 1.33)	0.9193	0.90 (0.66, 1.22)	0.4921	1.04 (0.74, 1.48)	0.8069
Q3	0.90 (0.66, 1.23)	0.5225	0.93 (0.69, 1.27)	0.6653	1.10 (0.77, 1.58)	0.6015
Q4	1.26 (0.91, 1.76)	0.1690	1.41 (1.01, 1.95)	0.0416	2.14 (1.46, 3.14)	< 0.0001
*P* for trend	1.13 (0.93, 1.39)	0.2209	1.24 (1.01, 1.52)	0.0368	1.61 (1.27, 2.03)	< 0.0001

Model 1 was adjusted for age, sex, race, education level, marital status, RIP, BMI, smoking status, alcohol consumption, hypertension, and diabetes

*OR* odds ratio, *95% CI* 95% confidence interval, *Ref* Reference

### Subgroup analysis

**Fig. 2:**
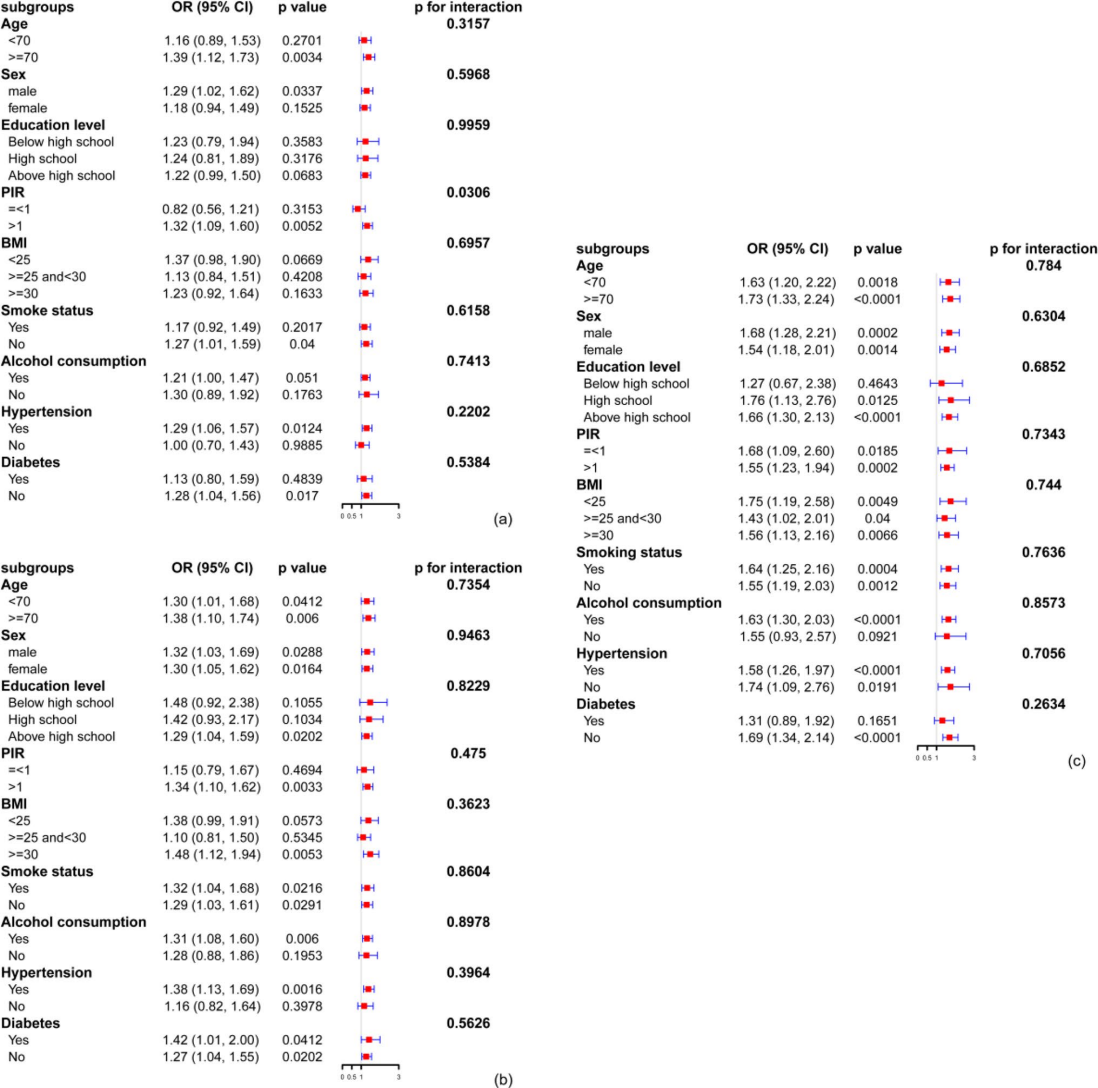
Subgroup analysis of associations between the WWI and low cognitive performance. Notes: **a** Associations between the WWI and low cognitive performance related to CERAD W-L. **b** Associations between the WWI and low cognitive performance related to AFT. **c** Associations between the WWI and low cognitive performance related to DSST

In the present study, to assess the stability of the correlation between the WWI and low cognition, we performed subgroup analyses and interaction tests stratified by age, sex, educational level, PIR, BMI, smoking status, alcohol consumption, hypertension and diabetes. The outcomes are displayed in Fig. 2. The results show that except for the PIR stratification (*p* = 0.03 for interaction) in the low cognitive performance group as indicated by CERAD W-L, other stratifications affecting the positive relationship between the WWI and low cognition were not significant (*p* > 0.05 for all interactions).

### Non-linear relationship between the WWI and low cognitive performance

**Fig. 3:**
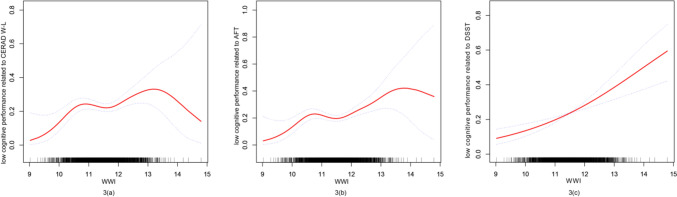
Smooth curve fitting for WWI and low cognitive performance related to CERAD W-L, AFT and DSST. Notes: The smoothed curve fit between the variables is shown by the red solid line. The area between the blue dashed lines indicates the fitted 95% confidence interval

In Model 3, there was a nonlinear relationship between the WWI and low cognition associated with CERAD W-L (Fig. 3a) and AFT (Fig. 3b). The threshold effect was calculated by fitting each interval using the segmented regression model (Table 3). For WWI and low cognitive performance associated with CERAD W-L, we detected an inflection point at 10.51. A positive correlation (OR = 5.36, 95% CI 1.48–19.34, *p* = 0.0104) was observed when the WWI was less than 10.51, while no correlation (OR = 1.11, 95% CI 0.92–1.35, *p* = 0.2574) was observed when the WWI was above this point. Like linear correlations, there is also a positive link between the WWI and poor cognition associated with AFT in the performance of nonlinear models. The breakpoint of WWI for low cognitive performance associated with AFT was 10.33, and low cognitive function associated with AFT was positively correlated whether the WWI was higher than 10.33 (OR = 7.71, 95% CI (1.19–50.05), *p* = 0.0324) or less than 10.33 (OR = 1.22, 95% CI (1.02–1.47), *p* = 0.0296). Furthermore, the smooth curve fit outcomes displayed a nonsignificant nonlinear association between the WWI and DSST-related low cognitive performance (Fig. 3c).

**Table 3:** Threshold effect analysis for correlations between the WWI and low cognitive performance

Outcomes	CERAD W-L	AFT
*Linear effect model*		
OR, (95% CI), *p* value	1.22 (1.03, 1.45) 0.0239	1.30 (1.09, 1.54) 0.0029
*Non-linear model*		
Inflection point (K)	10.51	10.33
OR1, (95% CI)(< K), *p* value	5.36 (1.48, 19.34) 0.0104	7.71 (1.19, 50.05) 0.0324
OR2, (95% CI)(> K), *p* value	1.11 (0.92, 1.35) 0.2574	1.22 (1.02, 1.47) 0.0296
LLR	0.013	0.037

*LLR* logarithmic likelihood ratio test *p* value

## Discussion

This research looked at the connection between the WWI and three tests of low cognitive function in US civilians. In this cross-sectional study which recruited 2762 individuals aged 60 years and over, the authors found a marked correlation between the WWI and low cognition, and this correlation was not significantly dependent on age, sex, race, education, BMI, smoke, drink, hypertension, or diabetes. In a model with all adjustments, a positive relationship was found between the WWI and poor cognitive function indicated by CERADW-L, AFT and DSST. Furthermore, further analysis showed a stable positive correlation between the WWI and low cognitive function indicated by the AFT and DSST.

Previous findings about the link between poor cognition and obesity-related parameters in older persons are controversial. Some studies have demonstrated that a lower BMI is linked to better cognitive function and that an increased BMI is linked to poor cognitive function later in life [9–12]. According to other research, having a higher BMI protects against cognitive decline, whereas having a lower BMI increases the likelihood of having poor cognitive performance [13–16]. Furthermore, the correlation between BMI and cognitive function varied significantly by race [34]. The link between WC and cognition is equally controversial, with research indicating that cognitive decline in Americans is inversely correlated with higher WC [35]. Meanwhile, a US cohort study showed that higher WC was connected with an accelerated rate of declining cognitive function, while BMI had no relationship with the risk of declining cognition [36]. This investigation focused on the connection between the WWI and poor cognition performance. The WWI can reflect weight-independent central obesity and is universally applicable to all races [22]. A cohort study discovered that BMI-adjusted WC correlated with the risk of declining cognition in older individuals. BMI-adjusted WC reflects body fat, particularly visceral adipose tissue. An increased risk of poor cognition was correlated with increased BMI-adjusted WC [23]. A longitudinal study using sagittal abdominal diameter (SAD) as a criterion for central obesity also indicated that central obesity raises the risk of dementia and appears to be unrelated to comorbidities such as diabetes and cardiovascular disease [37]. Similar findings from a different cohort study demonstrated that older adults with normal weight but abdominal obesity had a higher risk of dementia than those without abdominal obesity [38]. Therefore, the WWI is likely to be a more reliable indicator for the detection of cognitive decline than commonly used body composition indices such as BMI. This is consistent with our finding that low cognitive performance is positively related to the WWI. In the AFT and DSST for cognitive function, this relationship was independent of elements such as age, sex, smoke, drink and illness. The link between the WWI and cognitive impairment in older Americans was explored in this cross-sectional study for the first time. The link between cognitive function and central obesity is more clearly illustrated by the WWI than by BMI-adjusted WC, and it is easy to calculate and measure for clinical use. The results of this study suggest that in older adults, early screening for adverse WWI may be an appropriate tool to distinguish at-risk populations and that effective management of the WWI may delay cognitive decline.

The correlation between central obesity and low cognitive function can be explained by a number of biological mechanisms. Obesity is a chronic, persistent inflammatory state. Obesity increases the secretion of proinflammatory factors that trigger local inflammation in the brain and lead to neurodegeneration; at the same time, excess proinflammatory factors also lead to insulin resistance and thereby trigger metabolic syndrome, which is linked to low cognitive function and an elevated risk of dementia [7, 39–42]. Second, the above correlation may be related to hormones secreted by adipose tissue. Inflammation associated with obesity leads to leptin resistance and reduced secretion of lipocalin and induces neurodegeneration in the brain and the development of diseases associated with neurodegenerative disorders, including AD [43, 44]. Finally, obesity can damage the blood‒brain barrier and cause cerebrovascular dysfunction, which can affect cognitive performance [45, 46].

This research has several benefits. First, this research was founded on statistics from the NHANES, including population-based survey data with a significant societal impact that were gathered across the nation using a predetermined methodology. As a result of all analyses including the correct NHANES sampling weights, the research samples were more typical. The researchers performed subgroup analysis to verify stability and controlled for confounding variables to increase the credibility of the results. Finally, nonlinear relationships were also explored.

Using a larger sample capacity and adjusting for appropriate covariates, the reliability and representativeness of our findings were increased. However, the current research still has several flaws. First, as this was a cross-sectional study that looked at the link between the WWI and poor cognition in a large sample of people, a causal relationship between the two could not be determined. Therefore, to verify the causal link, prospective research with a larger sample size is needed. Second, even though the results were obtained from a nationally significant dataset, data on cognitive function were only available for the years 2011–2014. Therefore, in the future, further large-scale cohort studies may be needed to prove the current findings. Last, the sample for this research was solely based on U.S. older adults aged 60 years or older, so the applicability of the findings to the entire older population still needs to be investigated.

## Conclusion

This research shows that an increased risk of low cognitive function is associated with elevated WWI values, suggesting that clinical monitoring to detect a high WWI at an early stage and aggressive weight control can help prevent cognitive decline. More prospective research is needed in the future to prove the findings of this paper.

## Data Availability

The data used in this study are open to the public and the dataset is available at www.cdc.gov/nchs/nhanes/.
